# Competition between items in working memory leads to forgetting

**DOI:** 10.1038/ncomms6768

**Published:** 2014-12-18

**Authors:** Jarrod A. Lewis-Peacock, Kenneth A. Norman

**Affiliations:** 1 Department of Psychology and Imaging Research Center, University of Texas at Austin, Austin, Texas 78712, USA; 2 Department of Psychology and Princeton Neuroscience Institute, Princeton University, Princeton, New Jersey 08544, USA

## Abstract

Switching attention from one thought to the next propels our mental lives forward. However, it is unclear how this thought-juggling affects our ability to remember these thoughts. Here we show that competition between the neural representations of pictures in working memory can impair subsequent recognition of those pictures. We use pattern classifiers to decode functional magnetic resonance imaging (fMRI) data from a retro-cueing task where participants juggle two pictures in working memory. Trial-by-trial fluctuations in neural dynamics are predictive of performance on a surprise recognition memory test: trials that elicit similar levels of classifier evidence for both pictures (indicating close competition) are associated with worse memory performance than trials where participants switch decisively from thinking about one picture to the other. This result is consistent with the non-monotonic plasticity hypothesis, which predicts that close competition can trigger weakening of memories that lose the competition, leading to subsequent forgetting.

## Abstract (web summary)

Rapidly switching the focus of attention is believed to impair memory, but it is unclear how. Lewis-Peacock and Norman use brain imaging and multivariate analysis to show that when two memories briefly and closely compete in the brain, there is a lasting impairment in the ability to remember these thoughts.

## Graphical abstract

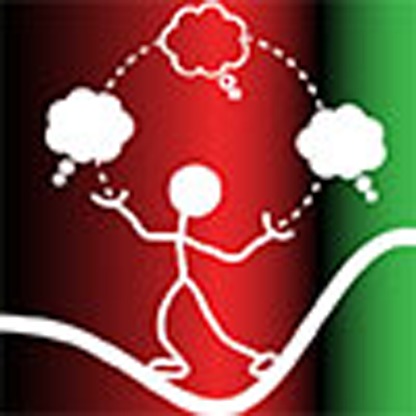

We constantly juggle our thoughts, and the activation of representations in working memory waxes and wanes over time according to the relevance of these representations. How does this juggling affect our ability to remember these items in the future? Specifically, can juggling thoughts in working memory do lasting harm to the representations of these thoughts in long-term memory?

Previous studies exploring inhibition of return phenomena have demonstrated that deactivating representations (of spatial locations^1^,^2^, pictures^3^ or task sets^4^,^5^) can lead to a short-term decrease (on the order of seconds) in participants’ ability to reactivate the previously attended representation. Here we explore whether—in some circumstances—there might be longer-term negative consequences of switching between thoughts. When people switch between thoughts under time pressure, the incoming thought and the outgoing thought will be co-active for some period of time (as the incoming thought’s activation is rising and the outgoing thought’s activation is falling), resulting in competition between these thoughts. In this study, we tested the prediction that competition between thoughts in working memory can harm subsequent memory of these thoughts.

This prediction follows from the non-monotonic plasticity hypothesis^6^,^7^, which posits a U-shaped relationship between memory activation and learning, such that moderate levels of memory activation lead to weakening of the memory, whereas higher levels of activation lead to strengthening (see Fig. 1, top). The non-monotonic plasticity hypothesis receives support from neurophysiological data showing that moderate postsynaptic depolarization leads to long-term depression (that is, synaptic weakening) and stronger depolarization leads to long-term potentiation (that is, synaptic strengthening)^8^,^9^,^10^. Recently, the non-monotonic plasticity hypothesis has also received support from human neuroimaging studies showing a U-shaped relationship between how strongly a representation comes to mind (measured using electroencephalography or functional magnetic resonance imaging (fMRI)) and the subsequent accessibility of that representation^6^,^7^,^11^. The non-monotonic plasticity hypothesis makes clear predictions regarding how competition should affect learning. Specifically, when memories compete, the ‘winning’ memory (that is, the memory receiving the most excitatory input) will be highly active, which should lead to further strengthening; ‘runner up’ memories (that is, memories receiving substantial excitatory input, but less than the winning memory) will end up being moderately active, which should lead to weakening of these memories; and memories that do not compete will not be strengthened or weakened^12^,^13^.

Figure 1 shows how thought-juggling can harm memories. Consider a situation where you are meeting a friend in an unfamiliar city. She texts you a photograph of a coffee house that is to be your rendezvous point. As you walk downtown, you think about this house and search for it on each new city block. You pull out your phone to text your friend, but discover that your phone has died. Now you start thinking about your friend’s face and hope that you will be able to recognize her in a bustling downtown. According to the non-monotonic plasticity hypothesis, subsequent memory for the coffee house will be a function of how decisively you switch back and forth between thinking about the coffee house and your friend’s face. If you switch decisively, minimizing the amount of time that the face and house compete, your house representation will spend relatively little time in the ‘weakening zone’ that leads to forgetting (see Fig. 1); hence, subsequent memory for the house will be relatively spared; conversely, if the house and face are thrust into prolonged competition (such that your house representation spends a higher amount of time in the ‘weakening zone’), then the house memory will be weakened, resulting in worse subsequent memory performance.

To test this prediction, we used a paradigm in which participants were presented with two pictures (a face and a scene) on each trial and were instructed to remember both, but to focus their attention on remembering the scene. Usually, scene memory was probed after a brief delay (stay trial), but occasionally (on one-third of the trials) a switch cue would appear instead, indicating that the face memory would be probed on this trial after another brief delay (switch trial). Based on prior work using a similar paradigm^14^,^15^,^16^,^17^, we hypothesized that participants would strongly activate a representation of the scene during the initial memory delay. When a switch cue was presented, we hypothesized that participants would deactivate their representation of the scene and activate their representation of the face. Our analysis focused on switch trials, which provide us with two opportunities to study competitive dynamics and learning: the pre-switch period, where participants are activating the scene representation, and the post-switch period, where participants are deactivating the scene representation. Stay trials were not ideally suited for testing our ideas about how competition affects subsequent memory; for discussion of stay trials and results from these trials, see Supplementary Note 1 and Supplementary Fig. 1.

The non-monotonic plasticity hypothesis predicts that close competition between the face and scene representations during the pre-switch or post-switch periods should be associated with worse subsequent memory for scenes, relative to trials where there is less competition. To test this prediction, we used pattern classifiers, applied to fMRI data in humans, to measure scene and face processing in the ventral temporal cortex (see Methods) throughout each trial^18^,^19^,^20^,^21^,^22^; we then related these neural measurements of scene/face processing (classifier evidence) to memory performance on a surprise recognition memory test for the scenes at the end of the experiment^23^,^24^,^25^. The ventral temporal regions that we used to measure scene/face processing themselves serve as inputs to convergence zones (for example, in the medial temporal lobes) that are responsible for storing long-term memories^26^; this means that we can treat our scene and face classifier evidence scores as reflecting the strength of the excitatory inputs into memory regions. As such, we can use the difference in scene and face classifier evidence (hereafter referred to as scene–face evidence) to track the ‘competitive balance’ between the scene and face, and use this to predict learning. In keeping with our theory, we find that moderate levels of scene–face evidence during switch trials (indicating close competition) are associated with worse subsequent recognition of scenes, relative to higher and lower levels of scene–face evidence. This result demonstrates, for the first time, that the manner in which we juggle our thoughts in working memory can have lasting, negative consequences.

## Results

### Behavioural results

Figure 2 shows the basic design of the experiment, which was composed of three phases: In Phase 1, participants performed a simple delayed-recognition task in the scanner; data from this phase were used to train the fMRI classifier. In Phase 2, participants performed the retro-cueing stay/switch task in the scanner. In Phase 3, participants were given a (behavioural) recognition test for scenes from Phase 2.

For the Phase 1 task in which participants performed simple delayed recognition of individual face and scene images, the mean response accuracy was 85.9% (s.e.m. 10.0%) and the mean response time was 511 ms (s.e.m. 22 ms), with no significant differences between face and scene trials (both *P*’s>0.63, two-tailed paired *t*-test). For the Phase 2 retro-cueing task in which participants performed delayed recognition of one image from an initial set of two, the mean response accuracy for switch trials was 85.1% (s.e.m. 2.5%) and the mean response time was 401 ms (s.e.m. 13 ms). Performance on the surprise recognition test in Phase 3 is shown in Fig. 2d. The mean hit rate for scenes previously studied in Phase 2 switch trials was 70.4% (s.e.m. 3.1%).

For all subsequent memory analyses of old items described below, we treated Phase 3 recognition responses as a graded measure of memory strength (sure old=1, unsure old=0.667, unsure new=0.333, sure new=0), in which the ‘old’ responses corresponded to remembered items and ‘new’ responses corresponded to forgotten items. Recognition memory sensitivity for scenes previously studied in Phase 2 switch trials was significantly above chance (two-tailed *t*-test on area under the receiver operating characteristic curve, *t*(20)=13.51, *P*<0.001; Fig. 2e).

### Measuring working memory dynamics

Group-averaged cross-validation results for the classifiers, trained separately on Phase 1 data for each participant, are shown in Fig. 3a. The cross-validation procedure entailed training a classifier on three blocks of data and then applying that classifier to independent data from the held out fourth block; the blocks were then rotated and this procedure was repeated until all four blocks had been tested. Face/scene decoding was well above chance. Scene evidence was reliably higher than face evidence for scene trials, and vice versa (both *P*’s<0.001), but scene and face scores were not dissociable during rest periods (*P*=0.58). To analyse data from Phase 2, classifiers were re-trained on all Phase 1 data, separately for each subject, before being applied to that subject’s Phase 2 data. Group-averaged classification results for switch trials (Fig. 3b) show that scene evidence was higher than face evidence throughout the initial delay period when participants were anticipating a memory probe of the scene target (two-tailed paired *t*-tests between 6 and 12 s; all *P*’s<0.0036, Bonferroni corrected for multiple time points). This relationship inverted following the switch cue such that face evidence was higher than scene evidence, and this difference persisted through the end of the trial (16–28 s; all *P*’s<0.0036). A recoded version of these data shows that on average, the scene-minus-face difference score (‘scene–face’) was positive during the pre-switch interval and negative during the post-switch interval, although there was extensive variability across trials (Fig. 3c). As noted above, we hypothesized that the difference between the strengths of competing memories would predict subsequent memory for scenes^6^,^7^,^11^,^12^,^13^.

### Relating classifier evidence to subsequent memory

For the analyses that follow, we computed average scene–face classifier evidence on each switch trial during an interval meant to capture pre-switch activity (4–12 s, not shifting for haemodynamic lag) and an interval meant to capture post-switch activity (16–20 s, not shifting for haemodynamic lag). The pre-switch interval was chosen to start 4 s after the beginning of the trial (to account for haemodynamic lag) and to end at the moment when the switch cue appeared. The post-switch interval was chosen to start 4 s after the switch cue appeared (to account for haemodynamic lag) and to end at the moment that the memory probe appeared.

We hypothesized that across items, there would be a non-monotonic (U-shaped) relationship between scene–face classifier evidence in Phase 2 and subsequent recognition memory for scenes in Phase 3. To formally test for the non-monotonic pattern in these data, we used the Probabilistic Curve Induction and Testing Toolbox (P-CIT) Bayesian curve-fitting algorithm^7^ to estimate the shape of the ‘plasticity curve’ relating working memory dynamics (indexed by scene–face classifier evidence) and subsequent memory performance for that scene. The P-CIT algorithm approximates the posterior distribution over plasticity curves (that is, which curves are most probable, given the neural and behavioural data). P-CIT generates this approximation by randomly sampling curves (piecewise-linear curves with three segments) and then assigning each curve an importance weight that quantifies how well the curve explains the observed relationship between neural and behavioural data. Finally, these importance weights are used to compute the probability of each curve, given the neural and behavioural data. To assess evidence for the non-monotonic plasticity hypothesis, P-CIT labels each sampled curve as theory consistent (if it shows a U shape, dropping below its starting point and then rising above its minimum value) or theory inconsistent, and then computes a log Bayes factor score that represents the log ratio of evidence for versus against the non-monotonic plasticity hypothesis; positive values of this score indicate a balance of evidence in support of non-monotonic plasticity. P-CIT also computes a *χ*^2^-test that assesses how well the curve explains the data overall, regardless of its shape; the *P*-value for this *χ*^2^-test indicates the probability of obtaining the observed level of predictive accuracy, under a null model where classifier evidence is unrelated to memory behaviour (see Supplementary Methods for additional justification of the P-CIT approach and a detailed description of how P-CIT works).

For our main P-CIT analysis, the pre-switch interval and the post-switch interval (for each scene item) were treated as separate learning events whose effects were summed to model recognition of that item. The fitted curves explained a significant amount of variance in subsequent recognition outcomes, *χ*^2^=18.89, *P*<0.0001. Most importantly, the curves recovered by P-CIT revealed a U-shaped mapping between classifier evidence scores and subsequent memory outcomes, such that moderate levels of scene–face evidence were associated with worse subsequent memory than higher and lower levels of scene–face evidence (log Bayes factor=2.4, Fig. 4a, top). This result is predicted by the non-monotonic plasticity hypothesis^6^,^7^,^11^,^12^,^13^.

To test the probability of getting this level of theory consistency due to chance, we estimated the null distribution by running a version of this analysis in which the mapping between the classifier evidence scores and the recognition data was permuted across trials. Only 1.5% of the permuted analyses that we ran (out of 200 total) showed log Bayes factors that matched or exceeded the log Bayes factor of the unpermuted data, indicating that it would be very unlikely to achieve this level of theory consistency due to chance.

To assess the population-level reliability of the U-shaped curve (that is, were the results driven by a small subset of participants), we also ran a bootstrap resampling test in which we resampled data from participants with replacement and re-computed the log Bayes factor for the resampled data. Ninety eight per cent of these bootstrap samples (out of 200 total) showed evidence in support of the non-monotonic plasticity hypothesis (that is, a positive log Bayes factor), thereby indicating a high degree of population-level reliability in the shape of the curve (Fig. 4b).

To assess whether the predicted U-shaped relationship between classifier evidence and memory was present in the pre-switch and post-switch periods (considered on their own), we ran the same P-CIT procedure described above, but separately on the pre-switch and post-switch data. The curves recovered by P-CIT based on pre-switch data (Fig. 4a, bottom) explained a significant amount of variance in subsequent recognition outcomes (*χ*^2^=13.82, *P*<0.001) and they revealed a U-shaped mapping between classifier evidence and memory outcomes that is consistent with our hypothesis (log Bayes factor=1.05). Permutation tests revealed that this level of theory consistency was unlikely to have occurred due to chance (only 3.5% of 200 permutations obtained a log Bayes factor greater than the observed log Bayes factor). Furthermore, 96% of 200 bootstrap samples showed evidence in support of the non-monotonic plasticity hypothesis (that is, a positive log Bayes factor), indicating a high degree of population-level reliability (Fig. 4b). Moving on to the analyses of post-switch data, the curves recovered by P-CIT based on post-switch data (Fig. 4a, bottom) also explained a significant amount of variance in subsequent recognition outcomes (*χ*^2^=10.34, *P*=0.001) and they were also U-shaped (log Bayes factor=1.81). Permutation tests revealed that this level of theory consistency was unlikely to have occurred due to chance (only 4.0% of 200 permutations obtained a log Bayes factor greater than the observed log Bayes factor). Furthermore, 96.5% of 200 bootstrap samples showed evidence in support of the non-monotonic plasticity hypothesis (that is, a positive log Bayes factor), indicating a high degree of population-level reliability (Fig. 4b).

One question that arises is whether pre-switch and post-switch processing make distinct U-shaped contributions to subsequent memory, or whether the U-shaped curve obtained post switch (or pre switch) is somehow an artefact of post-switch classifier evidence and pre-switch classifier evidence being correlated. To address this point, we ran analyses with P-CIT (see Supplementary Methods for details) to assess whether a U-shaped curve is obtained for the post-switch data after accounting for the predictiveness of the pre-switch data, and vice-versa. We found that there was still a significant predictive relationship between post-switch classifier evidence and subsequent memory after partialing out pre-switch classifier evidence (*χ*^2^=17.35, *P*=0.015), and the resulting curve still had a U shape (log Bayes factor=1.76; 98% of bootstraps had log Bayes factor>0). Likewise, there was still a significant predictive relationship between pre-switch classifier evidence and subsequent memory after partialing out post-switch classifier evidence (*χ*^2^=19.92, *P*=0.007), and the resulting curve still had a U shape (log Bayes factor=0.92; 91.5% of bootstraps had log Bayes factor>0). Permutation tests revealed that these levels of theory consistency were unlikely to have occurred due to chance. Only 3.5% of 200 permutations (for postswitch-partialing-out-preswitch) and 4.5% of 200 permutations (for preswitch-partialing-out-postswitch) obtained a log Bayes factor greater than the observed log Bayes factor.

Although the curves derived by P-CIT based on the pre-switch and post-switch intervals (separately) were both reliably U-shaped, they had a slightly different shape (Fig. 4a, bottom). These differences can be explained in terms of differences in the distributions of scene–face classifier evidence scores obtained for these intervals (Fig. 4c). The post-switch distribution is biased towards negative values (face dominance)—the right side of this distribution is associated with greater competition (that is, smaller absolute differences between scene and face evidence), which explains why the minimum value in the post-switch P-CIT curve (that is, the value corresponding to the worst subsequent memory performance) occurs towards the right side of this curve. Conversely, the pre-switch distribution is biased towards positive values (scene dominance)—the left side of this distribution is associated with greater competition, which explains why the minimum value in the pre-switch P-CIT curve occurs towards the left side of the curve.

### Relating classifier evidence to working memory performance

In addition to predicting subsequent memory performance, we also examined the relationship between working memory dynamics during Phase 2 switch trials and performance on the Phase 2 working memory probes (Fig. 5). Note that all of our claims about memory being a non-monotonic function of scene–face classifier evidence only apply to long-term memory modification. We expected that performance on the Phase 2 probe task would be a simple linear function of classifier evidence for the target category (on switch trials, face). Accordingly, we used a simple logistic regression analysis to assess the strength of the relationship between neural dynamics and performance on Phase 2 working memory probes. To validate that the shape of the relationship was truly linear (as predicted), we also ran P-CIT analyses on these data.

To maintain comparability with our analyses predicting Phase 3 performance, we measured working memory dynamics during the same time intervals in each trial (pre-switch 4–12 s, post-switch 16–20 s). We also analysed classifier evidence during a ‘probe’ window (20–24 s, not shifted to account for haemodynamic lag). Factoring in haemodynamic effects, this ‘probe’ window reflects processing that occurred before and during the onset of the probe. We expected that the predictive relationship between neural dynamics and probe performance would be highest for classifier measurements taken close in time to the probe (that is, 20–24 s) relative to classifier measurements taken earlier in the trial.

We found that during the pre-switch and post-switch intervals, neither face, scene, nor scene–face evidence scores were predictive of working memory accuracy (4–12 s, all *P*’s>0.16; 16–20 s, all *P*’s>0.08). During the probe period, however, stronger face evidence scores were associated with more accurate responses to the face probe (20–24 s, *P*=0.013); a similar trend was present for the relative face–scene scores (that is, a negative trend for scene–face; *P*=0.052). Lastly, follow-up analyses using P-CIT revealed that the function relating face evidence and working memory accuracy was monotonically increasing for the probe period (Fig. 5b).

## Discussion

**Figure 1:**
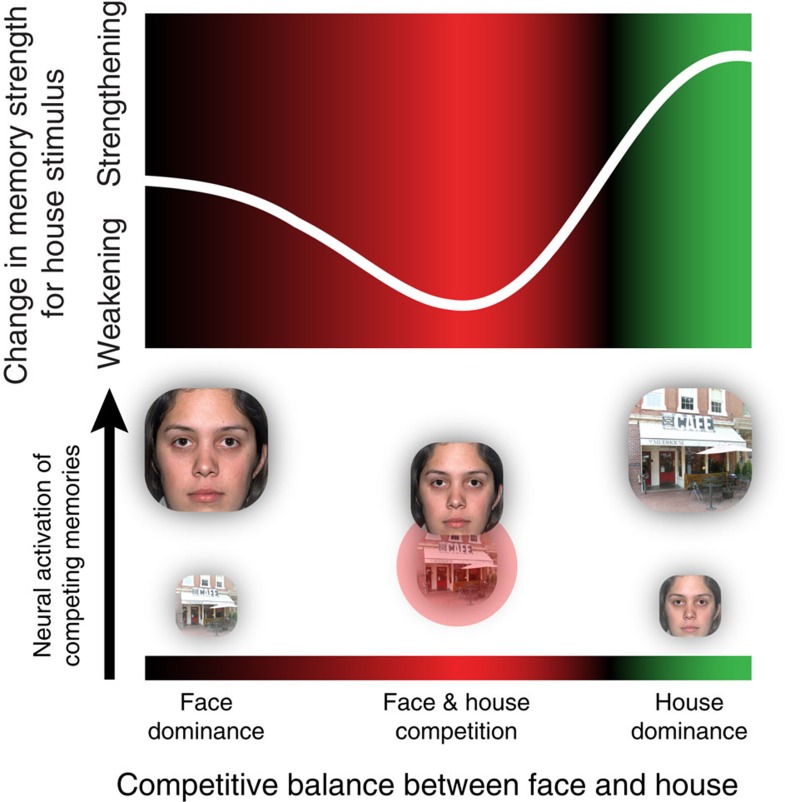
Hypothesized ‘plasticity curve’ describing how competition between memories drives learning. If a memory competes and clearly wins, it ends up being highly active and is strengthened; if the memory competes but does not win, it ends up being moderately active and is weakened; if the memory does not compete strongly, nothing happens. The background colour redundantly codes whether different levels of memory activation are linked to weakening (red) or strengthening (green). The diagram below the curve depicts different states of face/house competition that could occur during the rendezvous example in the text. When switching from the ‘house dominance’ state on the right to the ‘face dominance’ state on the left (or vice versa), the face and house pass through the ‘weakening zone’ of the plasticity curve where they are thrust into close competition with each other, resulting in moderate levels of house activity and (through this) weakening of the house memory. The greater the amount of time that the house spends in this ‘weakening zone’, the worse subsequent memory for the house will be.

The aim of the present study was to explore how juggling thoughts in working memory can impair subsequent memory for these thoughts. We used multivariate pattern analysis of fMRI data to demonstrate a U-shaped relationship between scene–face classifier evidence and subsequent memory for scenes. Compared with items that elicited relatively high or low levels of scene–face evidence (indicating scene or face dominance), items that elicited a moderate level of scene–face evidence (indicating close competition between the scene and face) were associated with worse subsequent memory performance. This relationship was present when we modelled recognition outcomes based on the sum of the effects of pre-switch and post-switch neural dynamics, and it was also present when we looked separately at pre-switch and post-switch dynamics. The shape of this plasticity curve fits with the non-monotonic plasticity hypothesis^6^,^7^, which predicts that close competition between memories will result in strengthening of the winning memory and weakening of competing memories (see Fig. 1). With regard to scene memory, if the scene representation wins (as indicated by a large positive value of scene–face evidence) the hypothesis predicts that it will be strengthened. If it clearly loses (as indicated by a large negative value of scene–face evidence), no learning is predicted to take place; it is specifically for intermediate values of scene–face evidence (where the scene competes but loses) that the non-monotonic plasticity hypothesis predicts weakening of the scene.

This study builds on other, recent neuroimaging studies that demonstrated a link between moderate levels of processing and memory weakening^6^,^7^,^11^. The key novel contribution of this study is a psychological one: the discovery that the manner in which we juggle our thoughts in working memory can have lasting, negative consequences on the subsequent accessibility of these thoughts. Although other studies have shown transient effects of thought-juggling (‘inhibition of return’ reductions in reaction time, lasting milliseconds or seconds^1^,^2^,^3^,^4^,^5^), ours is—to the best of our knowledge—the first study to show that inefficiently switching between thoughts in working memory can impair the subsequent accessibility of these thoughts (relative to trials where switching is more efficient) for at least several minutes. This finding has clear real-world consequences. Although thought-juggling (‘multi-tasking’) has been shown to degrade the quality of ongoing processing^27^, our study reveals that brief periods of competition between thoughts in working memory can do lasting harm to our ability to subsequently remember these thoughts.

Our analyses revealed significant and unique contributions to subsequent memory performance from the neural dynamics throughout the working memory trials. Rather than singling out one particular phase of thought-juggling as crucial for subsequent memory (for example, pre-switch: prioritizing one thought over another immediately after encoding, or post-switch: switching from one thought to another in working memory), our results highlight the importance of thought-juggling in general. Any time thoughts compete for neural resources in working memory, there is an opportunity for competition-dependent weakening of their representations in long-term memory. This finding has broad consequences, because this type of mental activity—thought-juggling—pervades our moment-to-moment mental lives. The necessary and sufficient conditions for competition to arise (for example, the relatedness of the co-activated thoughts and the duration of the thought-juggling process in working memory) are being tested in ongoing research.

In our experiment, there was no way to determine *a priori* which conditions would lead to moderate levels of scene–face evidence (thereby resulting in worse subsequent memory) and which conditions would lead to high levels of scene–face evidence (thereby resulting in better subsequent memory). The best we could do in this situation was to use conditions that trigger a range of scene–face values and then hope that these values sampled a wide-enough range to trace out the distinctive ‘U’ shape of the curve predicted by the non-monotonic plasticity hypothesis (that is, with increasing scene–face classifier evidence values, we should first see worse, then better subsequent memory performance). The ranges of scene–face evidence elicited during the post-switch and pre-switch periods were somewhat different (scenes were relatively dominant during the pre-switch period and faces were relatively dominant during the post-switch period); however, in both cases there was enough variance in scene–face evidence for the P-CIT algorithm to recover the predicted non-monotonic curve.

Importantly, the predicted U-shaped relationship between scene–face evidence and subsequent memory is specific to tests of long-term memory. On short-term memory tests that can be solved using active maintenance of the memory target, we predict a simple positive, linear relationship between classifier evidence for the target category and memory performance. In keeping with this prediction, we found a monotonically increasing relationship between target-category evidence and Phase 2 working-memory accuracy.

Although our preferred interpretation of the present results is that competitive neural dynamics cause weakening of the scene memory during the Phase 2 working memory task (and thus worse subsequent memory on the Phase 3 surprise memory test), it may also be possible to explain our results in terms of retrieval interference caused by learning of new associations during Phase 2 (for discussion of interference models and their possible application to ‘inhibitory’ memory phenomena, see ref. 28). If the face and scene representations are co-active during Phase 2, then (due to Hebbian synaptic plasticity) participants might form a new association between the face and scene representations. Later, when the scene is tested for recognition during Phase 3, the associated face might come to mind and interfere with retrieval of other information. The problem with this account is that it is unclear why face retrieval during Phase 3 would actually harm recognition judgments of the scene (to the contrary, face retrieval constitutes evidence that the scene was studied during Phase 2—otherwise, how would it have been associated with a face?). Tomlinson *et al*.^28^ specifically note that recognition tests are relatively immune to this kind of interference effect, compared, for example, with recall tests; as such, it seems unlikely that the observed decrement in Phase 3 recognition (for scenes eliciting moderate levels of scene–face evidence) would be attributable to interference at retrieval.

Another alternative account relates to trial-by-trial fluctuations in attention. According to this account, participants fail to attend on some trials, leading to (1) poor subsequent memory and (2) low levels of scene and face processing, which in turn will show up as moderate (near zero) scene–face evidence (zero scene−zero face=zero). This combination of factors could possibly explain the observed association between moderate scene–face evidence and poor subsequent memory. Crucially, this account predicts that scene and face evidence should both be low on trials leading to poor subsequent memory. We evaluated this prediction by plotting scene and face evidence as a function of subsequent memory (Supplementary Fig. 2). We found that contrary to the attention hypothesis, scene and face evidence values were reliably well above zero, even when subsequent memory was poor. This example does, however, highlight an important caveat regarding the use of scene–face evidence to predict subsequent memory. Specifically, scene–face evidence can only be viewed as a measure of competition in situations (similar to this experiment) where participants are reliably attending to the scene and/or face, resulting in above-floor levels of scene and/or face evidence. In situations where attention is fluctuating more strongly, scene–face values near zero might indicate inattention, which could lead to poor memory for completely different reasons.

This study has focused on situations where participants are switching between thoughts under time pressure, resulting in coactivity (and thus competition) between incoming and outgoing thoughts. This raises the question of whether competition of this sort is necessary to trigger forgetting. According to our model, the answer is no. The non-monotonic plasticity hypothesis posits that weakening occurs when a representation is moderately active. Competition between representations is one way to elicit moderate activity in memory regions, but there are other ways of eliciting moderate activity that have been shown to lead to worse subsequent memory: for example, briefly presenting a retrieval cue for a stored, paired associate^29^.

One final question is whether the learning mechanisms described here can be used in the service of deliberate (that is, motivated) forgetting. In our experiment, the forgetting effects observed on the Phase 3 memory test were an incidental byproduct of participants’ efforts to keep the appropriate stimulus in mind (that is, activated in working memory) during Phase 2. However, these learning mechanisms should also be operative in situations where people are asked to forget—in particular, they may be applicable to item-method-directed forgetting experiments, in which participants are instructed to deliberately forget certain stimuli immediately after encoding them. In this paradigm, successful processing of a forget instruction leads to a reduction in the availability of processing resources for subsequent items^30^,^31^. This suggests that the process of intentional forgetting may not result from passive decay, but rather from an active cognitive mechanism. The present results suggest that one mechanism that participants could use to achieve forgetting is to partially—but not completely—withdraw attention from the to-be-forgotten stimulus representation, thereby placing it in a situation where it competes with the new focus of attention (whatever that might be) and loses. Exploring whether participants actually do this is a future direction for our research.

In summary, we used pattern classification of fMRI data to show, for the first time, that close competition between representations in working memory can impair subsequent memory for the competing items. This shows a new, previously unappreciated ‘dark side’ to the constant thought-juggling that we do throughout the day. Every time that we activate a representation in working memory, it is at risk of being weakened as a result of competition with other representations. Fortunately, this research suggests how this memory loss can be avoided. By switching between thoughts cleanly, minimizing the amount of time that the incoming and outgoing thoughts come into contact with each other, we can maximize subsequent retention of these thoughts. It is a focus of our future research to determine the extent to which people have sufficient control over their thought-switching behaviour to either reliably induce forgetting (by increasing the amount of neural competition) or to reliably promote remembering (by reducing the amount of competition).

## Methods

### Participants

Twenty-one participants (11 female, aged 18–29 years, all right-handed) were recruited for this study using online scheduling software provided by the Department of Psychology at Princeton University. Sample size was chosen based on related studies from our lab^7^,^11^. Participants were compensated with $40 for their participation in the 2-h experiment. Written informed consent was obtained in a manner approved by the Princeton Institutional Review Board.

### Stimulus details

A large collection of face stimuli (including from www.macbrain.org/resources.htm^32^) and scene stimuli (including from http://cvcl.mit.edu/MM/sceneCategories.html^33^) was gathered through various online and in-house sources. A subset of these stimuli were chosen for this experiment based on memorability ratings from a stimulus evaluation experiment conducted through Amazon.com’s Mechanical Turk (see Supplementary Note 2). The final stimulus set consisted of 282 grayscale images of male and female faces cropped at the neck, and 282 grayscale images of indoor and outdoor scenes. Unique subsets of stimuli were used for targets in each of the first two phases of the experiment and no target stimulus was ever reused as a target or a probe in another trial within a phase. Sixty stimuli (30 faces and 30 scenes) were set aside in each phase for use as memory lures. The eight-picture probe display on each Phase 2 trial was constructed by sampling randomly (with replacement across trials) from the set of lures. The assignment of stimuli to experimental phases and (within phases) to the target and lure conditions was done randomly for each participant.

### Behavioural paradigm

**Figure 2:**
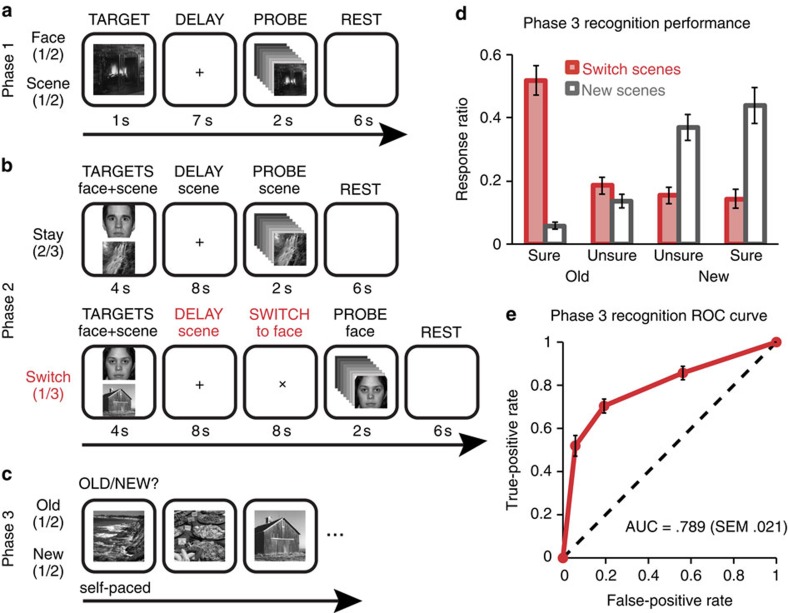
Task procedures and subsequent memory performance. (**a**) Participants performed delayed-recognition of a face or a scene picture during Phase 1. (**b**) Participants then performed retro-cued delayed recognition of one stimulus from a pair of target pictures (one face, one scene) during Phase 2. On 2/3 of the these trials, participants were tested on the scene (Stay trials); on 1/3 of the these trials, participants were given a switch cue at the end of the initial delay period, informing them that they would be tested on the face, not the scene (Switch trials). (**c**) In Phase 3 at the end of the experiment, participants were given a surprise memory test for scenes that were previously studied on Switch trials in Phase 2, and for new scenes. (**d**) Recognition confidence judgments for old and new scenes in Phase 3. (**e**) Recognition memory sensitivity as assessed by receiver operating characteristic (ROC) analysis for old scenes in Phase 3. AUC, area under the ROC curve. Error bars indicate the s.e.m., *n*=21.

**Figure 3:**
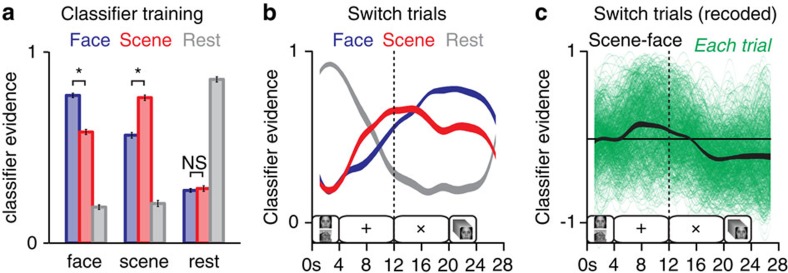
Pattern classification of fMRI data. (**a**) Classifier evidence scores for Phase 1 data, obtained by training the classifier on all but one Phase 1 block and testing on the remaining block. Face evidence is blue, scene evidence is red and resting-state evidence is grey (*P<0.001, face versus scene, paired *t*-test). (**b**) Trial-averaged decoding of switch trials from Phase 2, with evidence values interpolated between discrete data points every 2 s. Trial events are diagrammed along the horizontal axis. (**c**) Recoded classifier evidence scores (‘scene–face’) for switch trials overlaid on a distribution of single-trial traces from every participant. (Error bars and ribbon thickness indicate the s.e.m. across participants, *n*=21; see Supplementary Figs 2 and 3 for individual-subject versions of these plots). Note that in these plots classifier evidence scores were not shifted to account for haemodynamic lag.

**Figure 4:**
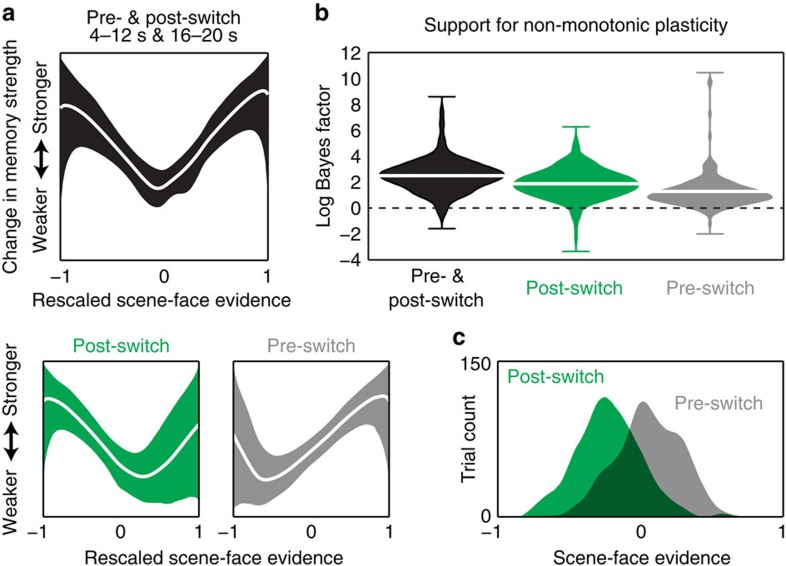
Relating classifier evidence on switch trials to subsequent recognition memory. (**a**) Empirically derived estimates (generated using the Bayesian P-CIT algorithm^7^) of the ‘plasticity curve’ relating scene–face evidence on switch trials to Phase 3 subsequent memory strength (operationalized as recognition confidence). Within each box, the line shows the mean of the posterior distribution over curves and the ribbon shows the 90% credible interval (such that 90% of the curve probability mass lies within the ribbon). The horizontal axis shows scene–face classifier evidence scores rescaled so that the minimum classifier evidence value=−1 and the maximum classifier evidence value=1; the vertical axis represents the change in subsequent memory strength. The box on the top shows the estimated curve when behavioural outcomes are modelled as depending on the summed effects of pre-switch (4–12 s) and post-switch (16–20 s) classifier evidence. The two boxes on the bottom show the estimated plasticity curve when behavioural outcomes are modelled as depending only on post-switch or pre-switch classifier evidence, respectively (*n*=21). (**b**) Violin plots describing the balance of evidence (operationalized in terms of log Bayes factor) in favour of the non-monotonic plasticity hypothesis, shown separately for the three analysis conditions. These plots show the probability density (using kernel density estimation) of the log Bayes factor derived from 200 bootstrap iterations for each analysis condition. The height of each plot indicates the full range of the data and the white marker indicates the mean. Positive values of the log Bayes factor correspond to evidence in favour of the non-monotonic plasticity hypothesis and negative values correspond to evidence against the hypothesis. (**c**) Histograms of scene–face classifier evidence during pre-switch (4–12 s; grey) and post-switch (16–20 s; green) intervals.

**Figure 5:**
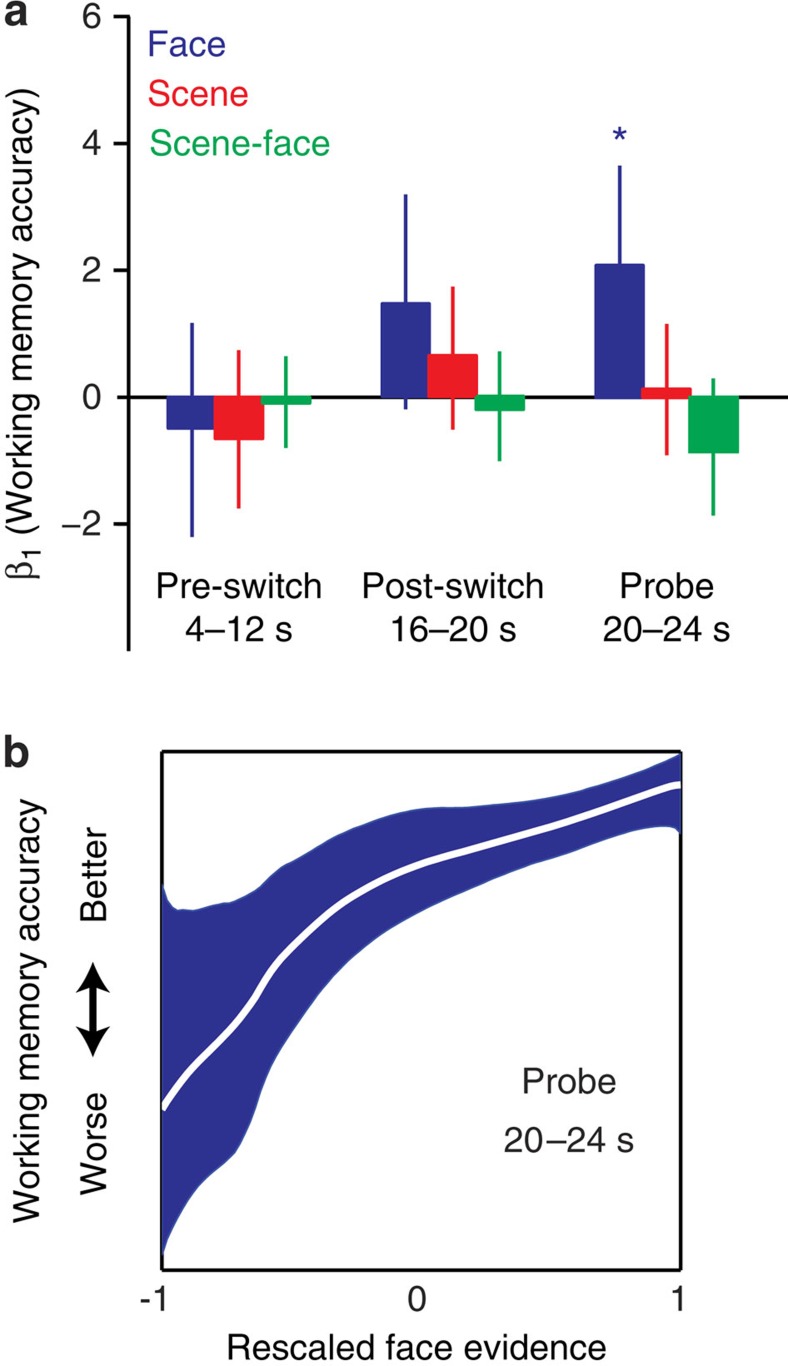
Relating classifier evidence on switch trials to working memory performance. (**a**) Logistic regression fit (*β*_1_) of face, scene and scene–face classifier evidence versus response accuracy on switch trials from the Phase 2 delayed-recognition task. The left group represents switch trials during the pre-switch interval (4–12 s), the middle group represents switch trials during the post-switch interval (16–20 s) and the right group represents switch trials during the probe interval (20–24 s). Error bars are 95% bootstrap confidence intervals. (*P<0.05, 1,000 bootstrap samples, *n*=21). (**b**) Empirically derived estimates (generated using the P-CIT algorithm^7^) of the curve relating face evidence during the 20–24 s probe window and working memory accuracy, showing a positive, monotonic relationship. The graph conventions are as described in Fig. 4.

The experiment proceeded in three phases (Fig. 2). In Phase 1, participants performed delayed recognition of face and scene pictures. Each trial began with a target display (1 s), followed by a fixation cross (7 s), a probe display (2 s) and a blank screen (6 s). All target displays consisted of one face or scene picture that appeared in the centre of the screen. After the target disappeared, participants were asked to maintain central fixation while they focused and sustained their attention on their memory for the target in preparation for a delayed-recognition probe. All probe displays consisted of a rapid serial visual presentation of eight pictures (drawn from the same category as the target) in the centre of the screen (125 ms each). The target reappeared in the probe stream on half of the trials (in rapid serial visual presentation position 3, 4, 5 or 6, chosen randomly) and lures were selected at random from a set of 30 face and 30 scene pictures that were never presented as targets. We used probe streams (rather than a single probe picture) to increase task difficulty and to encourage participants to sustain strong representations of the target during the delay period. Within a 1-s response window after the final probe picture, participants indicated with a yes/no button press whether the target was included in the probe stream.

In Phase 2, participants performed delayed recognition of face and scene pictures; here, two target pictures were presented on every trial but only one target was relevant for the recognition probe. Each trial began with a target display (4 s) followed by a fixation cross (8 s). All target displays consisted of one face and one scene picture, one of which appeared on the top half of the screen and the other appeared on the bottom half (the category order was counterbalanced across trials). Participants were instructed to form a separate mental image for each picture and not to imagine the face and scene interacting in any way. After the targets disappeared, participants were to maintain central fixation while they focused and sustained their attention on their memory for the scene target, in preparation for a delayed-recognition probe of that scene. On two-thirds of trials (‘stay’ trials), the delay-period fixation cross was followed by a probe display (2 s) and then a blank screen (6 s). However, on the other one-third of trials (‘switch’ trials), the fixation cross rotated 45° and remained on the screen for an additional 8 s before the onset of the probe display. This rotated cross served as a retro-cue for participants to switch their focus of sustained attention away from their memory of the scene target and towards their memory of the face target, in preparation for a delayed-recognition probe of that face. The ratio of switch trials to stay trials was set at 1:2 rather than, for example, 1:1, to encourage participants to prioritize retention of the scene at the beginning of every trial. The probes were configured similarly to the Phase 1 probes, except that there were an equal number of face and scene pictures in each probe stream (125 ms each; four faces and four scenes, randomly ordered). For stay trials, the scene target reappeared in the probe stream in half of the trials; the face target reappeared in the probe stream on every trial as a memory lure. For switch trials, the face target reappeared in the probe stream in half of the trials; the scene target never reappeared in the probe stream (we did this to avoid repetition-based memory enhancement of the scene). On all trials, participants indicated with a yes/no button press, within a 1-s response window after the final probe picture, whether the appropriate target was included in the probe stream.

Finally, at the end of the experiment (in Phase 3), participants were presented with a surprise old/new recognition memory test for the scene pictures that were presented as targets during the Phase 2 trials. In the test, 72 previously viewed scenes (36 from switch trials; 36 from stay trials) were randomly interleaved with 72 novel scenes. Note that all of the tested scenes from Phase 2 appeared exactly once during that phase (at the start of the trial); if a scene also appeared in the probe at the end of a stay trial, it was excluded from testing. Each image remained on the screen until participants responded with one of four responses on the button box (1: sure old; 2: unsure old; 3: unsure new; 4: sure new). This memory test was administered inside the scanner ~5 min after participants completed the final trial of the Phase 2 task. As a result, the average amount of time that elapsed between initial exposure of a scene in Phase 2 and its subsequent exposure in Phase 3 was ~30 min.

Our design is deliberately asymmetric. The experiment was set up to induce participants to focus on scenes during the pre-switching period and faces during the post-switch period (never the other way around) during Phase 2, and during Phase 3 we tested scene memory but not face memory. We introduced this asymmetry because of prior evidence from our lab^7^ and other labs^34^ that fMRI pattern classifiers are more sensitive to scene processing than face processing. We reasoned that to the extent that scenes generate a higher-fidelity neural readout, this could give us the extra resolution required to discriminate between theory-consistent (U-shaped) curves and theory-inconsistent curves.

### fMRI data collection

The experiment was presented using Psychophysics Toolbox Version 3 in Matlab running on a Mac Pro. The Phase 1 task was divided into four 20-trial blocks (5 min 40 s each) with an even number of face and scene trials in each block. The Phase 2 task was divided into six 18-trial blocks (7 min 8 s each) with 12 stay trials and 6 switch trials in each block. Total functional scanning time for the first two phases was 65 min 28 s. All blocks were preceded by 20 s of dummy pulses to achieve a steady state of tissue magnetization. Between blocks, participants were given a break during which the experimenter checked that the participant was comfortable and alert. Whole-brain images were acquired with a 3T MRI scanner (Siemens Skyra). First, we ran a brief scout localizer scan (15 s) to verify that head position was within the designated field of view and to derive automatic anterior commissure–posterior commissure alignment parameters for subsequent scans. For Phases 1 and 2, we used a gradient-echo, echo-planar sequence (repetition time=2,000 ms, echo time=34 ms), with automatic shimming enabled, to acquire T2*-weighted data sensitive to the blood-oxygen-level dependent signal within a 64 × 64 matrix (196 mm FoV, 34 axial slices, 3 mm^3^ isotropic voxels) using integrated parallel acquisition techniques with both retrospective and prospective acquisition motion correction enabled. Finally, we used a magnetization-prepared rapid gradient-echo (MPRAGE) sequence to acquire high-resolution T1-weighted images (repetition time=2,300 ms, echo time=3.08 ms, 0.9 mm^3^ isotropic voxels, 9 min 0 s acquisition time), while the participants performed the final Phase 3 recognition test in the scanner.

### fMRI preprocessing

Preprocessing of the functional data was done with the AFNI^35^ software package using the following preprocessing steps (in order): (1) we corrected for slice time acquisition with 3dTshift, (2) we rotated oblique data to cardinal direction with 3dWarp, (3) we resampled to a 3 mm^3^ gridset with 3dresample and (4) we realigned to the first volume of the Phase 1 data using rigid body alignment with 3dvolreg. Anatomical data were aligned to the first volume of the functional data with align_epi_anat.py. A whole-brain voxel mask was created for each participant by combining the results of 3dAutomask (dilation=1) across all ten functional runs.

To create anatomically derived region-of-interest (ROI) masks for each participant, an atlas-space transformation (AFNI’s TT_icbm452 atlas) was computed for the anatomical data of each participant with @auto_tlrc. Then, a ventral temporal cortex ROI was constructed for each participant by combining voxels from the TT_Daemon atlas mask for the full, bilateral fusiform and parahippocampal gyri and backward transforming that combined mask into the participant’s native space and intersecting it with that participant’s whole-brain mask. The mean number of voxels retained in this ventral temporal mask was 3,224 (s.d.=366). A feature selection analysis of variance was applied to the preprocessed fMRI data within the ventral temporal mask to select those voxels whose activity varied significantly (*P*<0.05) between face trials, scene trials and rest periods over the course of the Phase 1 task. The mean number of voxels passing feature selection was 969 (s.d.=216). The pattern of activity across these feature-selected voxels was used as the input to the pattern classifier. No spatial smoothing was imposed on the data and the data were analysed in each participant’s native space.

### Multi-voxel pattern analysis

Our goal in analysing the fMRI data was to obtain the most sensitive possible measure of face and scene processing. To accomplish this goal, we used multi-voxel pattern analysis (MVPA^18^,^19^,^20^,^21^,^22^) to decode face and scene processing, based on voxels from a ventral temporal ROI (as defined in the ‘fMRI preprocessing’ section above). For additional justification of the particulars of our MVPA approach, see Supplementary Note 3.

Pattern classifiers were trained, separately for each participant, on data from Phase 1. Specifically, classifiers were trained on individual brain scans (acquired as 2-s intervals) from the final 6 s of the delay period in each Phase 1 trial, plus data from the 6-s intertrial intervals. For the selected scans, one classifier was trained to distinguish between scans corresponding to the active retention of a face and other scans; another classifier was trained to distinguish between scans corresponding to the active retention of a scene and other scans; and a third classifier was trained to distinguish between periods of rest and other scans. Resting data were randomly sampled so that within each block of trials, the classifiers were trained on the same number of exemplars for all three categories (120 total scans each of face, scene and rest across all four Phase 1 blocks). As is standard practice in MVPA^22^, all trial regressors were shifted forward in time by 6 s to account for haemodynamic lag of the blood-oxygen-level dependent signal (typically estimated as 4–8 s to peak after event onset). We evaluated classifier training accuracy by using the method of *k*-fold cross-validation on the Phase 1 data, that is, training on *k*−1 blocks of data and testing on the *k*^th^ block and then rotating and repeating until all blocks had been classified. For each individual 2-s scan within a test block, the three classifiers each produced an estimate (from 0 to 1) of the degree of neural evidence for the category they were trained to detect (face, scene and rest). For decoding of the Phase 2 data, we trained the classifiers on all four blocks of Phase 1 data and then applied the classifiers to all six blocks of Phase 2 data to produce classification scores for every 2-s interval throughout the Phase 2 task. In addition to the category-specific evidence scores computed by the classifiers, we also calculated the difference between the scene and face evidence scores (that is, ‘scene–face’) at every time point. The continuous decoding of data from Phase 2 trials allowed for a complete characterization of evidence for face and scene processing in each trial.

All pattern classification analyses were performed using the Princeton MVPA Toolbox^36^ in Matlab (downloadable from http://www.pni.princeton.edu/mvpa), using L2-penalized logistic regression. The L2 regularization term biases the algorithm to find a solution that minimizes the sum of the squared feature weights. Logistic regression uses a parameter (*λ*) that determines the impact of the regularization term. To set the penalty *λ*, we explored how changing the penalty affected our ability to classify the Phase 1 data (using the cross-validation procedure described above). We found that the function relating *λ* to cross-validation accuracy was relatively flat across a wide range of *λ*-values (spanning from 0.001 to 1,000). We selected a *λ*-value in the middle of this range (*λ*=50) and used it for all of our classifier analyses. Note that we did not use the Phase 2 fMRI data in any way while selecting *λ* (otherwise, we would be vulnerable to concerns about circular analysis^37^ when classifying the Phase 2 data).

### Relating classifier evidence to subsequent memory

Our primary analysis goal was to evaluate the relationship between classifier evidence scores from the Phase 2 switch trials and recognition memory outcomes in Phase 3. To accomplish this goal, we first computed (separately for each Phase 2 switch trial) the average levels of face and scene classifier evidence both before and after the onset of the switch cue. For the pre-switch time window, we averaged together classifier evidence values from the entire first delay period, from 4 to 12 s of each trial (unadjusted for haemodynamic lag). For the post-switch time window, we averaged together classifier evidence values from 16 to 20 s of each trial (unadjusted for haemodynamic lag).

For our primary analysis, we estimated the shape of the ‘plasticity curve’ relating scene–face classifier evidence scores and recognition memory outcomes using the P-CIT Bayesian curve-fitting algorithm^7^. As noted in the Results section, P-CIT estimates (in a continuous manner) the posterior distribution over plasticity curves; it also allows us to compute a log Bayes factor score that represents the log ratio of evidence for versus against the non-monotonic plasticity hypothesis. We present a detailed description of the P-CIT analyses (as well as additional justification for these analyses) in the Supplementary Methods.

### Statistical procedures for assessing reliability

When analysing behavioural data (without respect to neural data) and neural data (without respect to behavioural data), we used standard random-effects statistics (paired *t*-tests, with subjects as a random effect) to assess the reliability of our results across participants.

For our analyses relating neural data (from Phase 2) to behaviour (in both Phase 2 and Phase 3), we combined individual trial data from each participant into one giant ‘supersubject’ and subsequently performed all statistical analyses on these amalgamated data^7^,^11^. We used this approach, chosen *a priori*, instead of the conventional random-effects approach (used elsewhere in the paper) in which the average results from each subject are used for group-level hypothesis testing. The reason for using the supersubject approach here is that we did not collect enough data from each individual participant to reliably estimate the relationship between neural data and memory behaviour within each participant. This problem was exacerbated by the fact that participants did not distribute their memory responses evenly across memory response bins; as a result, some response bins did not contain any observations within a particular participant and other bins only contained a handful of observations, leading to very noisy estimates of classifier evidence associated with that response bin (see Supplementary Figs 2 and 3 for individual-subject data). The problem was further exacerbated by the fact that (in our analyses relating Phase 2 neural data to Phase 3 behaviour) we were looking for nonlinear effects, which require much more data to estimate than linear effects.

In this experiment, each participant (*N*=21) contributed 36 samples of switch-trial data (a total of 756 trials). To assess population-level reliability of the results (that is, were they driven by a small subset of participants) from each of our analyses, we also ran a bootstrap test where we resampled data from participants with replacement and re-computed the analyses for this resampled data^38^. The population-level reliability of the results was reflected in the proportion of bootstrap samples in which the effect of interest (for example, log Bayes factor>0) was present. If this proportion is large, it indicates that the observed curve shape reflects a general property of the population being sampled and is not being driven by a small subset of participants.

In summary, the ‘supersubject+bootstrap’ approach taken here (in relating Phase 2 neural data to Phase 2 and Phase 3 behavioural data) allowed us to derive overall estimates of brain–behaviour relationships despite having noisy and/or incomplete data within individual subjects. It is important to emphasize that our supersubject+bootstrap approach—such as standard random effects analyses—permits inferences about population-level reliability of results (that is, if we collected another sample of participants from the same population, would we get the same effect?). The key difference between our approach and the standard random-effects approach is that (unlike random-effects analyses) our result does not permit inferences about whether the effect is reliably observed within individual subjects. We did not have enough data (relative to the level of noise in the data) to answer this question.

## Author contributions

J.A.L.-P. and K.A.N. designed the experiment. J.A.L.-P. collected the data and performed analyses. Both authors discussed the results and contributed to the writing of the manuscript.

## Additional information

**How to cite this article**: Lewis-Peacock, J. A. and Norman, K. A. Competition between items in working memory leads to forgetting. *Nat. Commun.* 5:5768 doi: 10.1038/ncomms6768 (2014).

## Supplementary Material

Supplementary Information Supplementary Figures 1-3, Supplementary Notes 1-3, Supplementary Methods and Supplementary References
