# Supplementary material for: Competition between items in working memory leads to forgetting
Source: Nat Commun. 2014 Dec 18;5:5768. doi: 10.1038/ncomms6768 (PMC4284654; doi:10.1038/ncomms6768)
Supplement: Supplementary Information — Supplementary Figures 1-3, Supplementary Notes 1-3, Supplementary Methods and Supplementary References [file ncomms6768-s1.pdf]

Supplementary Materials for:
Competition Between Items in Working Memory Leads to Forgetting

Jarrod A. Lewis-Peacock[1] and Kenneth A. Norman[2]

[1]*Department of Psychology and Imaging Research Center*
University of Texas at Austin, Austin, TX 78712, USA

[2]*Department of Psychology and Princeton Neuroscience Institute*
Princeton University, Princeton, NJ 08540, USA

CONTENTS

1. Supplementary Figures
    1.1. Supplementary Figure 1
    1.2. Supplementary Figure 2
    1.3. Supplementary Figure 3
2. Supplementary Notes
    2.1. Supplementary Note 1: Behavioral and neural results for stay trials
    2.2. Supplementary Note 2: Mechanical Turk for stimulus filtering
    2.3. Supplementary Note 3: Justification of multi-voxel pattern analysis methods
3. Supplementary Methods
4. Supplementary References

Email addresses: jalewpea@utexas.edu (Jarrod A. Lewis-Peacock), knorman@princeton.edu (Kenneth A. Norman)

SUPPLEMENTARY FIGURES

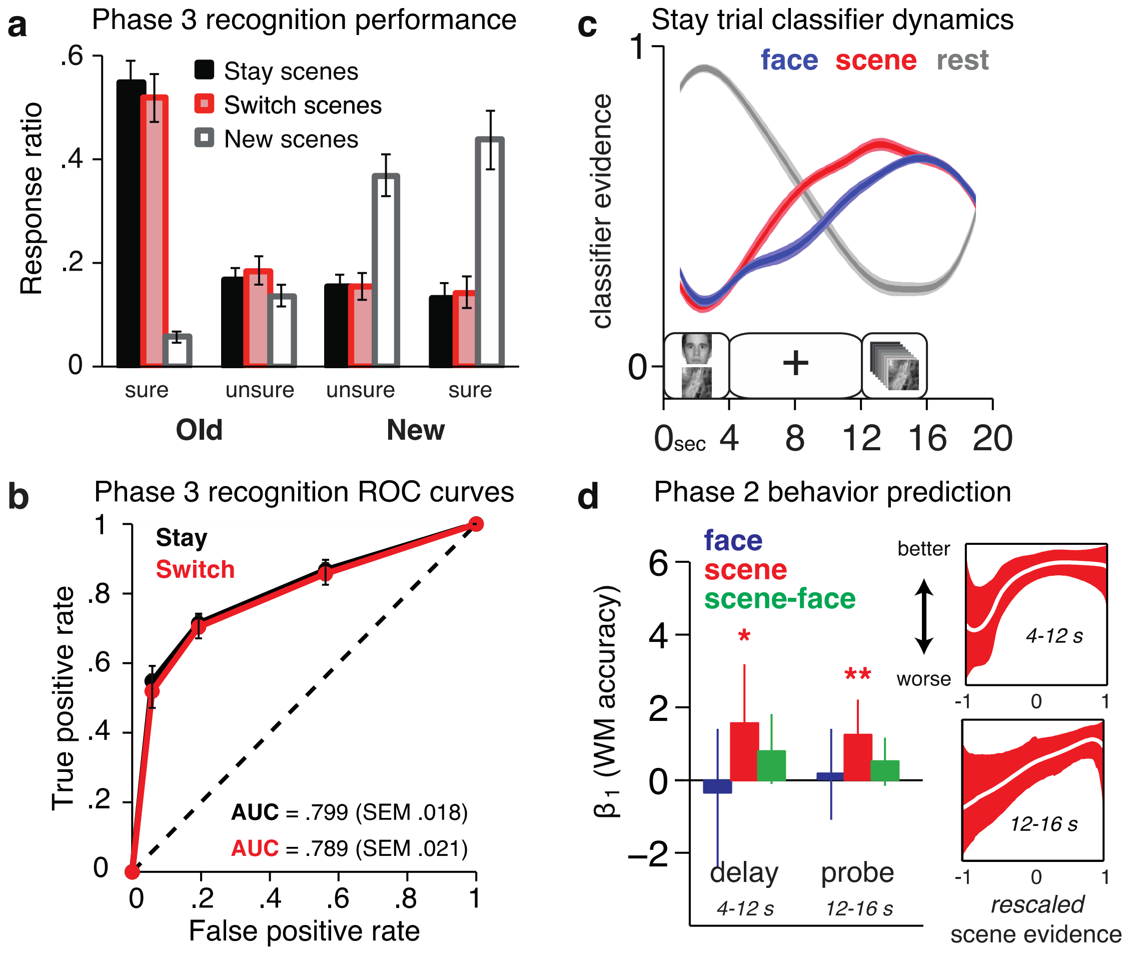

Supplementary Figure 1. Stay-trial behavioral and neural data (n = 21; for methods and discussion, see Supplementary Note 1). (A) Recognition judgments for scenes that were previously studied in Phase 2, and for a set of new scenes. (B) Recognition memory sensitivity as assessed by receiver operating characteristic (ROC) analysis for stay- and switch-trial scenes in Phase 3 (AUC = area under the ROC curve). Note that there is no statistical difference between recognition memory sensitivity for stay- and switch-trial scenes. (C) Trial-averaged classifier decoding of stay trials from Phase 2, with evidence values interpolated between discrete data points every 2 s. Trial events are diagrammed along the horizontal axis. Note that classifier evidence scores were not shifted to account for hemodynamic lag. (D) Logistic regression fit (β_1) of face, scene, and scene - face classifier evidence vs. response accuracy on the Phase 2 working memory task. These data represent stay trials during the delay period (4-12 s) prior to the onset of the probe, and during the probe window (12-16 s). Error bars are 95% bootstrap confidence intervals. (* $p < .05$, ** $p < .005$; 1,000 bootstrap samples). Empirically derived estimates (generated using the Bayesian P-CIT algorithm[1]) of the curve relating scene evidence to Phase 2 (working memory) response accuracy are shown for the delay period and the probe period.

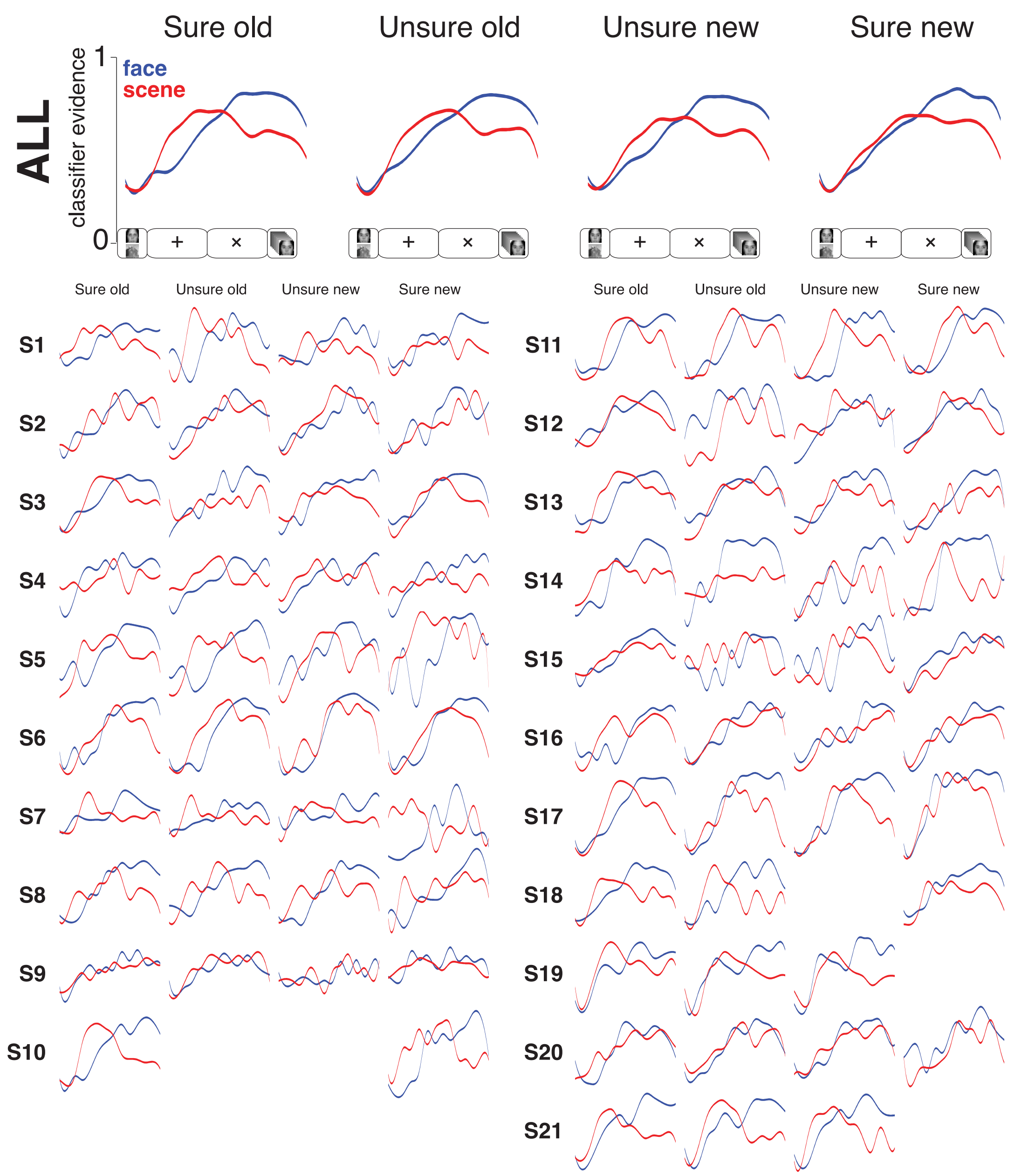

Supplementary Figure 2. Classifier evidence scores (scene and face) for each participant; evidence values were interpolated between discrete data points every 2 s (see Figure 3B in the main paper for these same results, collapsed across memory confidence and averaged across participants). The top row shows pooled data from each participant. The trial events are diagrammed along the bottom of the four graphs in this row. The remaining rows (split across two main columns) show data from individual participants. Within each of the two main columns, the first column represents trials in which the scene was later recognized with high confidence (a "sure old" response was given on the final surprise recognition test). The next three columns represent trials associated with "unsure old", "unsure new", and "sure new" responses. Note that, in these plots, classifier evidence scores were not shifted to account for hemodynamic lag. Qualitatively, the plots support the idea that closer competition (during both the pre-switch and post-switch period) lead to lower subsequent memory confidence. For detailed statistical analyses relating classifier evidence to subsequent memory confidence, see the P-CIT curve fitting results in the main paper.

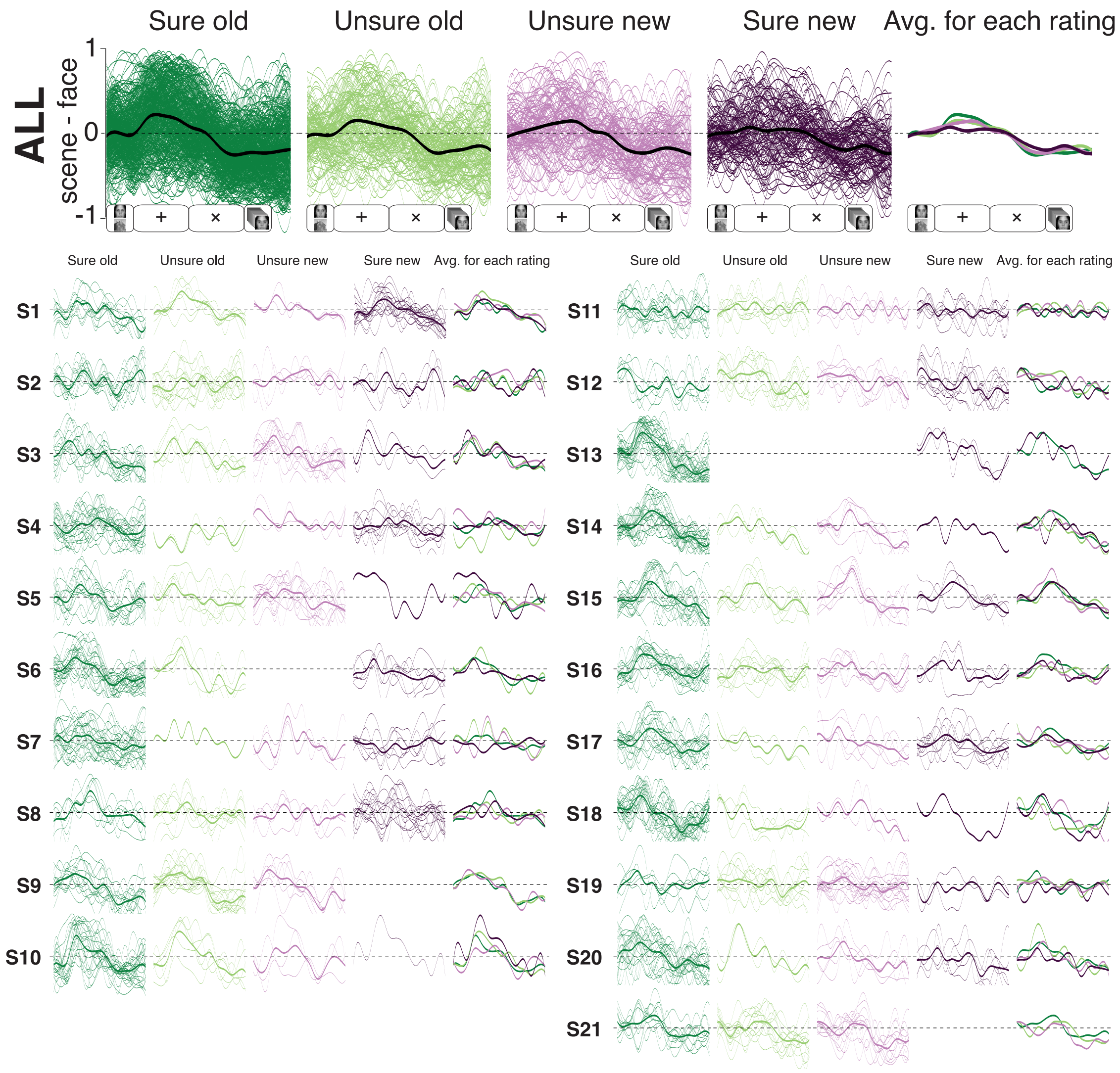

Supplementary Figure 3. Classifier evidence scores (scene - face) for each trial and each participant; evidence values were interpolated between discrete data points every 2 s (see Figure 3C in the main paper for these same results, collapsed across memory confidence and averaged across participants). The top row shows pooled data from each participant. The trial events are diagrammed along the bottom of the five graphs in this row. The remaining rows (split across two main columns) show data from individual participants. Within each of the two main columns, the first column (dark green) represents trials in which the scene was later recognized with high confidence (i.e., a "sure old" response was given on the final surprise recognition test). The next three columns represent trials associated with "unsure old" (light green), "unsure new" (light purple), and "sure new" (dark purple) responses. The final column shows the average classifier evidence trajectories for each level of response confidence for that participant. In each graph, the thin spaghetti lines represent classifier data for individual trials, and the thicker line represents the average classifier evidence trajectory for that response type for that individual. Note that, in these plots, classifier evidence scores were not shifted to account for hemodynamic lag.

SUPPLEMENTARY NOTES

Supplementary Note 1: Behavioral and neural results for stay trials

The purpose of including stay trials in our design was to engender appropriate cognitive dynamics during switch trials. Given that 2/3 of trials were stay trials (in which the scene target was tested by the working memory probe), we hypothesized that participants would be motivated to actively maintain the scene during the initial part of the trial; on trials where they received a switch cue, participants would switch from thinking about the scene to thinking about the face, thereby leading to competition that (according to our theory) would lead to memory weakening.

Importantly, while stay trials were useful for engendering appropriate cognitive dynamics on switch trials, the stay trials themselves were not ideally suited for testing our hypotheses regarding how neural dynamics affect subsequent scene memory. Like switch trials, stay trials contained an initial 4-12 s period where scene and face processing could be monitored and related to subsequent memory for the scene. However, unlike switch trials, stay trials contained a probe period at the end of the trial where memory was tested for the target scene. It is reasonable to think that participants would continue to actively maintain their representation of the target scene during the probe period, and that scene processing during the probe period would be consequential for subsequent memory for that scene. For example, maintaining a strong expectation of the target scene might lead to strengthening of the memory, and maintaining a moderate expectation might lead to weakening of the memory. However, unfortunately, we could not track processing of the target scene during the probe period: Because multiple scenes and faces were presented (in rapid succession) during the probe, it was impossible to attribute scene classifier evidence during the probe period to the target scene as opposed to one of the

other scenes. This inability to track processing of the target scene during a highly relevant part of the trial makes stay trials suboptimal for testing our hypotheses about how neural dynamics relate to subsequent memory. Nonetheless, for completeness, behavioral and fMRI analyses of stay-trial data are described below (with results shown in Supplementary Figure 1).

Note that the same issue (i.e., inability to track processing of the target scene during the probe period) could, in principle, also affect switch trials. However, it should be less of a concern, insofar as participants were motivated to keep the target *face* in mind during the probe period on switch trials (not the scene). Given that there was substantially *less processing of the target scene* during the probe period on switch (vs. stay) trials, we expected that the probe period would have less of an effect on subsequent memory for the target scene on switch (vs. stay) trials. We should also note that, if processing of the target scene *did* occur during the probe period on some switch trials, this would act as a source of noise in our analyses relating pre-switch and post-switch processing to subsequent memory (not a systematic bias).

Stay trial Phase 2 behavioral results

For the Phase 2 retro-cueing task in which participants performed delayed-recognition of one image from an initial set of two, the mean response accuracy for all stay trials was 81.9% (s.e.m. 2.5%) and the mean response time was 426 ms (s.e.m. 12 ms). Response accuracy on stay trials was not significantly different from accuracy on switch trials ($p > .07$). Participants responded more quickly on switch trials compared to stay trials ($t(20) = 3.12$, $p = .005$).

Stay trial Phase 3 behavioral results

During Phase 3, we only tested recognition memory for scenes from a subset of stay trials — specifically, stay trials where the target scene *did not appear* during the probe period at the

end of the trial. Supplementary Figure 1 (panels A and B) shows subsequent memory results for this subset of stay trials (along with results for switch trials and for new scenes presented only during Phase 3). Recognition memory sensitivity for scenes previously studied in Phase 2 stay trials was significantly above chance (two-tailed t-test on AUC, $t(20)=16.57$, $p<.001$). There was no significant difference between recognition memory sensitivity for stay trials and switch trials (two-tailed paired t-test on AUC, $t(20)=1.05$, $p=0.31$), nor were there any significant differences in the proportions of responses at each of the four memory confidence levels between the trial types (all p's > .18). Receiver operating characteristic (ROC) analyses were performed on the Phase 3 recognition memory data by calculating hit rates and false-alarm rates using thresholds corresponding to the different confidence levels. The five points of the ROC curve were calculated at different thresholds by: (1) considering all items as "old"; (2) considering all items as "old" except for "sure new" responses; (3) considering only "sure old" and "unsure old" as "old" items; (4) considering only "sure old" responses as "old" items; and (5) considering all items as "new". These hit rates and false-alarm rates were used to determine the area under the ROC curve (AUC) for each subject. A group t-test comparing the observed AUC to a chance-level of AUC=0.5 assessed the statistical reliability of the memory sensitivity scores separately for stay and switch trials, and a paired t-test was used to assess the difference between the two trial types.

A priori, one might expect recognition memory to be worse for switch trials than stay trials (because of the additional opportunities for competition — and thus competition-dependent weakening — on switch trials). One possible explanation for the lack of a significant difference relates to the fact (noted above) that recognition memory was only tested for stay trials where the target scene did not appear during the probe period. On these trials, participants were likely expecting the target scene to appear during the probe period; it is possible the competition

between this mental representation of the (expected) target scene and other non-target scenes during the probe period led to weakening of the target scene representation, thereby pulling down subsequent recognition memory performance for scene targets from stay trials.

Predicting Phase 3 recognition based on Phase 2 dynamics

Group-averaged classification results for stay trials (Supplementary Figure 1C) show that scene evidence was higher than face evidence throughout the delay period when participants were anticipating a memory probe of the scene target (two-tailed paired t-tests between 6-14 s; all p's < .005, Bonferroni corrected for multiple time points). We used the P-CIT algorithm to model the relationship between scene - face classifier evidence from the delay period of Phase 2 stay trials (4-12 s, not shifting for hemodynamic lag — this is the "pre-switch" time window from our analysis of switch trials) and Phase 3 recognition memory. In contrast to switch trials (where we found a significant predictive relationship between pre-switch classifier evidence and subsequent memory, as indicated by the χ^2 statistic from the likelihood ratio test), the relationship between stay-trial delay-period classifier evidence and subsequent memory was not significant ($\chi^2 = 1.931$, $p = .167$). This null result is likely due to the fact that additional learning was taking place on stay trials during the probe period in Phase 2, but we were unable to track processing related to the target scene during this period.

Relating classifier evidence to working memory performance

In addition to predicting subsequent memory performance, we also examined the relationship between processing dynamics during Phase 2 stay trials and performance on the Phase 2 working memory task (Supplementary Figure 1D, left). As discussed in the main paper, all of our claims about memory being a nonmonotonic function of scene - face classifier

evidence only apply to *long-term memory modification.* We expected that performance on the Phase 2 probe task would be a simple linear function of classifier evidence for the target category (on stay trials, scene). Accordingly, we used a simple logistic regression analysis to assess the strength of the relationship between neural dynamics and performance on Phase 2 working memory probes. To validate that the shape of the relationship was truly linear (as predicted) we also ran P-CIT analyses on these data. To maintain comparability with our analyses predicting Phase 3 performance, we looked at the same delay-period interval (4-12 s, not shifted to account for hemodynamic lag). We also measured classifier evidence during the "probe" interval (12-16 s, not shifted to account for hemodynamic lag). Factoring in hemodynamic effects, this "probe" window reflects processing that occurred immediately before and during the onset of the probe. We expected that the predictive relationship between neural dynamics and probe performance would be highest for classifier measurements taken close in time to the probe (i.e., 12-16 s) relative to classifier measurements taken earlier in the trial.

For stay trials (where the scene target was tested at the end of the delay period), scene evidence scores during the delay period (4-12 s) and the probe period (12-16 s) were predictive of working memory accuracy ($p = .029$ and $p = .004$, respectively); a similar trend was present for the relative scene - face scores during the probe period ($p = .051$). None of the other combinations of evidence type (scene, face, scene - face) and time window were significant. Follow-up analyses using P-CIT revealed that the function relating scene evidence and working memory accuracy was monotonically increasing for both the delay period and the probe period (Supplementary Figure 1D, right).

Supplementary Note 2: Mechanical Turk for stimulus filtering

Prior to running our main experiment, we sought to filter out stimuli that were judged to be too memorable or not memorable enough. Our intention was to reduce the influence of stimulus properties on Phase 3 recognition memory performance. To accomplish this, we collected normative ratings of our stimuli using Amazon.com's Mechanical Turk, a crowdsourcing internet marketplace available as part of Amazon Web Services. Mechanical Turk can reduce the time and cost of data collection while dramatically increasing sample sizes[2]. Based on prior work showing that the typicality and familiarity of an image can contribute to its overall memorability[3-5], we collected subjective ratings of these qualities for each of our images.

Task Design

A large collection of male and female faces, and indoor and outdoor scenes (1,008 total; 672 faces) was gathered through various online and in-house sources. These images were divided into groups of 25 images. The assignment of an image to a group (and the order of images within a group) was randomized. Each unit of work (a "human intelligence unit", or "HIT") for a Mechanical Turk participant consisted of 25 pages, where each page consisted of 1 image followed by a list of 6 questions:

1. "What type of image is this?" Choices: female face, male face, indoor scene, or outdoor scene." (If a user answered this objective question incorrectly, that user's responses to the remaining questions were excluded from the analysis.)

2. “In the space below, please categorize the image a little more specifically (for example, it could be a young dark-skinned woman or a beach). Your answer will be scrutinized, so you want to be as accurate as possible.”

3. “Relative to the category you described in Question 2, how typical is this image? (1-very atypical, 6-completely typical)”

4. “Relative to the other images you might see in the category you described above, how fast did you “take in” this image? (1-Relatively fast, 6-Relatively slowly)”

5. “How memorable is this image? (1-not memorable at all, 6-very memorable)”

6. “How familiar does this face or scene look? (1-very unfamiliar, 6-very familiar)”

The HIT was titled “Image Evaluation.” Its description was, “Evaluate various features of pictures of faces and scenes.” The keywords assigned to the HIT were “picture, face, scene, evaluate.” Participants provided informed consent via a “qualification quiz” mechanism provided by Mechanical Turk (http://mturk.s3.amazonaws.com/CLT_Tutorial/UserGuide.html) before they were allowed to view our task. Workers were allotted a maximum of 5 minutes to complete each image evaluation, and were allotted a maximum of 7 days to complete all images within a HIT.

Participants

A total of 1,009 unique “Mechanical Turk workers” participated in this study. We required workers to have a prior HIT approval rate greater than or equal to 95%, and approved all work completed by our workers (regardless of whether we subsequently excluded their data). We paid workers \$0.02 per image evaluation, or \$0.50 for each 25-image HIT. The average

response time per image evaluation was 45 s, and thus the average hourly rate for each HIT was $1.60.

Results

We received a total of 25,634 image evaluations, and excluded 5,679 of these evaluations (22.15%) for the following reasons: incorrect identification of the image category in Question #1 (651); an overly generic, repetitive, answer for the image description in Question #2 (925); no written description of the image in Question #2 (395); an overly fast response time that was at least 1 standard deviation faster than the mean (548); identical responses for Questions #3, #5, & #6, despite inverted scales on Questions #3 and #6 with respect to their hypothesized relationship to memorability (3,160). This left us with 19,955 total responses, or approximately 20 evaluations per image.

A composite "memorability" score was created for each image by averaging together the average typicality rating (Question #3) and the average memorability rating (Question #5) across all valid responses. Before being combined with the memorability ratings, the typicality ratings were recoded such that a high score corresponded to an "atypical" rating (atypical images have been linked to higher memorability[3-5]). The answers on the other subjective rating questions (Questions #4 and #6) did not correlate with the memorability question (Question #5) and therefore were excluded from further analysis. The average composite memorability score for faces was 3.61 (S.D. 0.15), and for scenes was 3.62 (S.D. 0.22). The 282 images from the middle of each distribution of scores were selected for use in the main experiment (selected faces spanned ratings of 3.53 to 3.68; selected scenes spanned ratings of 3.32 to 3.94).

Supplementary Note 3: Justification of multi-voxel pattern analysis methods

In analyzing the Phase 2 data, we used multi-voxel pattern analysis (MVPA[6-10]) to track face and scene processing, based on voxels from a ventral temporal ROI. Here, we provide additional justification for our MVPA approach.

Prior work has shown that multi-voxel pattern classifiers provide a more sensitive readout of category-specific processing than simply tracking the activations of peak category-selective regions like the fusiform face area and parahippocampal place area[11-12]; this increase in sensitivity is primarily attributable to classifiers' ability to exploit category information conveyed by voxels within ventral temporal cortex (but outside of peak category-selective regions[13]). The approach of limiting the classifier to ventral-temporal cortex (as opposed to the whole brain) results in a further boost in classification, by focusing the classifier on regions that we know (from prior work[13]) to be richly informative about visual categories. This ventral-temporal-masking procedure has been used in several other MVPA studies from our lab and others that have tracked scene and/or face processing[1, 14-18]. More generally, the use of category classifiers to track memory retrieval has become a standard approach in the memory literature (see[19] for a review).

A very large number of prior studies (from our lab and others) have observed a relationship between category classifier evidence and behavioral indices of item memory[1, 13-18, 20]. The logic of using category evidence to track item memory relies on the following ideas: First, thinking about an item should also activate the representation of the item's category (e.g., if you think of a specific scene, that will also activate the neural representation of "scenes" more generally); hence, in most situations, item and category processing should covary. Second, in our experiment, a specific scene and a specific face are presented at the start of each trial. While

participants could, in principle, be thinking of other faces/scenes during the pre-switch and post-switch intervals, they have no incentive to do this, so we treat face/scene classifier evidence during this trial as reflecting thoughts about the target face and the target scene. It is of course possible that face/scene classifier evidence on a given trial could reflect thoughts about a non-target face/scene, or generic thoughts about faces/scenes (in the absence of a specific scene representation); but if this were always the case, it would be impossible to explain the observed relationships between classifier evidence and memory behavior (e.g., the observation that face classifier evidence during the 20-24 sec interval predicts Phase 2 working memory accuracy on switch trials).

SUPPLEMENTARY METHODS

As noted in the main text, we used the P-CIT Bayesian curve-fitting algorithm[1] to estimate (in a continuous fashion) the shape of the "plasticity curve" relating Phase 2 scene - face classifier evidence scores and Phase 3 recognition memory outcomes. We chose this approach in an attempt to maximize our sensitivity for detecting non-monotonic relationships in the data. We considered the alternative approach of "binning" based on classifier evidence, and then comparing subsequent memory performance for the different bins; the problem with binning is that it imposes rigid and arbitrary boundaries between measured levels of classifier evidence, and thus it would reduce our ability to detect non-monotonic patterns in the data if they do not happen to fall along the boundaries defined by the bins.

For this particular application of P-CIT, pre-switch (4-12 s) and post-switch (16-20 s) trial intervals were treated as distinct learning events that could both affect subsequent memory. P-CIT generates a posterior probability distribution over plasticity curves via the following steps:

First, the algorithm rescales the predictor variable (here, scene - face classifier evidence) so the maximum observed value = 1 and the minimum observed value = -1. Next, the algorithm defines a parameterized family of curves (piecewise-linear curves with three segments) and randomly samples 100,000 curves from this parameterized space. For each one of the randomly sampled curves, we used that curve, coupled with pre- and post-switch scene - face classifier evidence values, to generate predictions about which scenes would be remembered or forgotten. Specifically, for each item, we separately computed the expected effect of pre-switch dynamics (by taking the measured level of scene - face evidence and evaluating the sampled plasticity curve at that value) and the expected effect of post-switch dynamics (by taking the measured level of scene - face evidence and evaluating the sampled plasticity curve at that value). To estimate the probability that the item would be remembered or forgotten, we summed the expected effects of pre- and post-switch dynamics and fed this sum into a linear function (the parameters of which were estimated by the model), which gave us an estimated probability of successful recognition for that scene item.

For each sampled curve, we compared these estimated probabilities of successful recognition (for each item) to the actual recognition outcomes, and assigned an importance weight to the curve reflecting how well the estimated recognition outcomes fit with the actual outcomes. This importance weight value summarizes how well that particular curve explains the observed relationship between neural data (i.e., classifier measurements of scene and face processing) and behavioral data (i.e., high/low confidence judgments about whether the previously viewed scene items are old or new).

After assigning importance weights to the 100,000 samples (using the procedure outlined above), the next step in the curve estimation process was to generate a new set of samples, according to the following procedure: First, we sampled (with replacement) from the existing set

of curves according to their importance weights, such that curves with large importance weights were selected more often. Second, for each (re-)sampled curve, we slightly distorted the parameters of the curve. These two steps were repeated 100,000 times so as to generate 100,000 new samples. This procedure had the effect of concentrating the samples in regions of curve parameter space that were associated with large importance weights. After generating these new samples, we alternated between (1) assigning importance weights to these new samples, and (2) resampling based on the new importance weights. In total, we ran the procedure for 10 iterations of generating samples and then assigning importance weights.

The collection of 100,000 weighted curves generated by this process can be interpreted as an approximate posterior probability distribution over curves – such that the weight of a curve is proportional to its probability. To generate a mean predicted curve, we averaged together the sampled curves in the final generation of samples, weighted by their importance values (see Fig. 4A in the main paper). We also computed credible intervals to indicate the spread of the posterior probability distribution around the mean curve. We did this by evaluating the final set of sampled curves at regular intervals along the x-axis (i.e., scene - face classifier evidence). For each x coordinate, we computed the 90% credible interval by finding the range of y values that contained the middle 90% of the curve probability mass. Note that, for this application of P-CIT, we were able to estimate the shape of the plasticity curve, but we were not able to estimate whether a given level of classifier evidence was associated with memory strengthening or weakening relative to a no-learning baseline (because our experiment did not include a "baseline" condition of this sort; see [1] for further discussion of this issue).

To summarize the level of evidence in favor of (or against) the non-monotonic plasticity hypothesis (i.e., that the true plasticity curve is U-shaped), we computed the log Bayes factor: the log of the ratio of the evidence *in favor of* our hypothesis to the evidence *against* our

hypothesis[21]. Positive values of the log Bayes factor indicate evidence in favor of non-monotonic plasticity, and negative values indicate evidence against non-monotonic plasticity.

We used the following procedure to compute the log Bayes factor: First, we labeled each sampled curve as theory-consistent or theory-inconsistent. Curves were labeled as theory-consistent if they showed a "dip" — i.e., the curve dropped below its starting point and then rose above its minimum value (moving from left to right). We then calculated the proportion of posterior probability mass taken up by theory-consistent samples, by summing together the importance weights associated with theory-consistent samples, and we calculated the probability mass taken up by theory-inconsistent samples, by summing together the importance weights associated with these samples. Finally, the log Bayes factor was computed as:

(1) $$f = ln\left[\left(\frac{\text{sum of importance weights for theory-consistent curves}}{\text{sum of importance weights for theory-inconsistent curves}}\right) \div \text{correction factor}\right]$$

where:

(2) $$\text{correction factor} = \frac{\text{proportion of the space of possible curves that is theory-consistent}}{\text{proportion of the space of possible curves that is theory-inconsistent}} = \frac{0.583}{0.417} = 1.398$$

The correction factor compensates for the fact that more than 50% (58.3%, to be precise) of the space of possible curves is theory-consistent, so we would expect a slight imbalance in favor of theory-consistency due to chance.

To assess whether the log Bayes factor values that we obtained (for the actual data) could have arisen due to chance, we ran nonparametric *permutation tests* for all of our P-CIT analyses relating Phase 2 classifier evidence to Phase 3 recognition memory. For these tests, we permuted the relationship between classifier evidence values and memory outcomes across trials (within a given "supersubject" — see *Statistical Procedures for Assessing Reliability*, in the *Methods*

section of the main paper). This permutation instantiates the null hypothesis that no relationship was present between the neural and behavioral data. Specifically, we permuted the data in this fashion 200 times; for each permutation, we re-ran the entire P-CIT procedure and re-computed the log Bayes factor. The resulting 200 log Bayes factor values served as an empirical null distribution for log Bayes factor (i.e., this is the distribution that we would expect if there were no real relationship between brain activity and behavior). By measuring where our actual log Bayes factor value fell on this distribution, we were able to compute the probability of getting this value or higher under the null hypothesis.

Lastly, P-CIT toolbox also automatically runs a *likelihood ratio test* to diagnose whether the curve-fitting analysis is modeling any signal in the data other than random noise. This likelihood ratio test is associated with a χ^2 value. The greater the amount of variance (in memory behavioral outcomes) explained by the plasticity curve, the greater the χ^2 value will be, and the smaller the associated *p* value will be (indicating decreasing probability of obtaining the observed level of predictive accuracy, under a null model where classifier evidence is unrelated to memory behavior). The likelihood ratio test can be viewed as “permissive” for the theory-consistency analyses outlined above — it only makes sense to evaluate the shape of the fitted curve if that curve actually explains behavioral variance (as indicated by a sufficiently low p value for the χ^2 test). The Matlab code used to perform the P-CIT analyses (along with detailed documentation) can be downloaded from http://code.google.com/p/p-cit-toolbox.

Separately Analyzing Pre-Switch and Post-Switch Data

In addition to modeling the summed effects of pre-switch and post-switch dynamics on subsequent recognition of scenes, we also ran follow-up analyses to assess whether the predicted U-shaped relationship between classifier evidence and memory was present in the pre-switch and

post-switch periods (considered on their own). To do this, we ran the same P-CIT procedure described above, but separately on the pre-switch and post-switch data. Note that, if pre-switch and post-switch classifier values are correlated, it is possible that P-CIT results obtained for pre-switch data could be an artifact of patterns that are present in post-switch data, and vice-versa. To guard against this possibility, we also ran an analysis using P-CIT to control for the effects of pre-switch classifier evidence on post-switch results, and vice-versa. Below, we describe the variant where we partial pre-switch classifier evidence out of the post-switch results; we also ran a variant where we partialed post-switch classifier evidence out of the pre-switch results.

To control for the effects of pre-switch dynamics on post-switch P-CIT results, we first used pre-switch classifier evidence to derive (using P-CIT) a mean predicted curve that (for the pre-switch data) best describes the relationship between scene - face evidence and whether the scene items were later remembered or forgotten. We then used the mean predicted curve to generate a predicted memory outcome value for each trial (i.e., for each trial, we took the pre-switch classifier evidence value as the x-axis value, and we looked up the corresponding y-coordinate on the curve). We then ran a linear regression where (across trials) we used "predicted memory outcome" values to predict the actual memory outcome for that trial (high-confidence forget = 0, low-confidence forget = 0.333, low-confidence remember = 0.667, high-confidence remember = 1). Finally, we took the residuals from this regression (reflecting variance unexplained by the pre-switch data), and we ran a second P-CIT analysis where we used the post-switch classifier evidence on each trial (as measured by the classifier) to predict the residual memory scores. To assess theory consistency, controlling for effects of pre-switch classifier evidence, we computed the log Bayes factor measure using the results of this second analysis.

SUPPLEMENTARY REFERENCES

1. Detre, G. J., Natarajan, A., Gershman, S. J. and Norman, K. A. Moderate levels of activation lead to forgetting in the think/no-think paradigm. *Neuropsychologia* **51**, 2371-88 (2013).

2. Kittur, A., Chi, E. H., Suh, B. Crowdsourcing User Studies With Mechanical Turk. *Proceeding of the 26th annual SIGCHI conference on human factors in computing systems*, New York, NY, 453-456 (2008).

3. Bartlett, J.C., Hurry, S., & Thorley, W. Typicality and familiarity of faces. *Mem. & Cogn.* **12**, 219-228 (1984).

4. Vokey, J. R. and Read, J. D. Familiarity, memorability, and the effect of typicality on the recognition of faces. *Mem. & Cogn.* **20**, 291-302 (1992).

5. Light, L.L., Kayra-Stuart, F., & Hollander, S. Recognition memory for typical and unusual faces. *J. Exp. Psychol. Hum. Learn.* **5**, 212-228 (1979).

6. Norman, K. A., Polyn, S. M., Detre, G. J., and Haxby, J. V. Beyond mind-reading: multi-voxel pattern analysis of fMRI data. *Trends Cogn. Sci.* **10**, 424-430 (2006).

7. Haynes, J. D., and Rees, G. Decoding mental states from brain activity in humans. *Nat. Rev. Neurosci.* **7**, 523-534 (2006).

8. Pereira, F., Mitchell, T., and Botvinick, M. Machine learning classifiers and fMRI: a tutorial overview. *Neuroimage* **45**, S199-S209 (2009).

9. Tong, F., and Pratte, M.S. Decoding patterns of human brain activity. *Annu. Rev. Psychol.* **63**, 483-509 (2012).

10. Lewis-Peacock, J. A. & Norman, K. A. Multi-voxel pattern analysis of fMRI data. In M.S. Gazzaniga & G.R. Mangun (Eds.), *The Cognitive Neurosciences*, **4th ed**. MIT Press, Cambridge, Massachusetts, USA (2014).

11. Lewis-Peacock, J. A., and Postle, B. R. Decoding the internal focus of attention. *Neuropsychologia* **50**, 470-478 (2012).

12. Polyn, S. M., Natu, V. S., Cohen, J. D., & Norman, K. A. Category-specific cortical activity precedes retrieval during memory search. *Science*, **310**, 1963-1966 (2005).

13. Haxby, J. V. *et al.* Distributed and overlapping representations of faces and objects in ventral temporal cortex. *Science* **293**, 2425-2430 (2001).

14. Kim, G., Lewis-Peacock, J. A., Norman, K. A. and Turk-Browne, N. B. Pruning of memories by context-based prediction error. *Proc. Natl. Acad. Sci. USA* **111**, 8997-9002 (2014).

15. Poppenk, J. and Norman, K. A. Briefly cuing memories leads to suppression of their neural representations. *J. Neurosci.* **34**, 8010-8020 (2014).

16. Kuhl, B. A., Rissman, J., Chun, M. M., & Wagner, A. D. Fidelity of neural reactivation reveals competition between memories. *Proc. Natl. Acad. Sci. USA*, **108**, 5903-5908 (2011).

17. Kuhl, B. A., Rissman, J., and Wagner, A. D. Multi-voxel patterns of visual category representation during episodic encoding are predictive of subsequent memory. *Neuropsychologia* **50**, 458-469 (2012).

18. Zeithamova, D., Dominick, A. L., & Preston, A. R. Hippocampal and ventral medial prefrontal activation during retrieval-mediated learning supports novel inference. *Neuron*, **75**, 168-179 (2012).

19. Rissman, J. and Wagner, A. D. Distributed representations in memory: insights from functional brain imaging. *Annu. Rev. Psychol.* **63,** 101-128 (2012).

20. Kuhl, B. A., Johnson, M. K. and Chun, M. M. Dissociable neural mechanisms for goal-directed versus incidental memory reactivation. *J. Neurosci.* **33,** 16099-16109 (2013).

21. Kass, R. E. and Raftery, A. E. Bayes factors. *J. Am. Stat. Assoc.* **90**, 773-795 (1995).
